# Implementation Research to Strengthen Health Care Financing Reforms Toward Universal Health Coverage in Indonesia: A Mixed-Methods Approach to Real-World Monitoring

**DOI:** 10.9745/GHSP-D-18-00328

**Published:** 2018-12-27

**Authors:** Rena Eichler, Susan Gigli, Lisa LeRoy

**Affiliations:** a Broad Branch Associates, Washington, DC, USA.; b Broad Branch Associates, Washington, DC, USA. Now with Kantar Public, Washington, DC, USA.; c Abt Associates, Cambridge, MA, USA.

## Abstract

Universal health coverage reforms are complex and impact numerous processes, institutions, and individuals. To know whether reforms are being implemented as planned and working as envisioned, policy makers and managers need information and insights on bottlenecks. The United States Agency for International Development (USAID) provided support to build implementation research (IR) capacity and to conduct cycles of research to help Indonesia understand how its single-payer national insurance reform, Jaminan Kesehatan Nasional (JKN), was affecting primary care. Two cycles of IR in Indonesia focused on effects of JKN financing on primary care, as determined through a consultative process with stakeholders at national and district levels. This process generated questions relevant for implementers and policy makers and strengthened government interest in findings. Research was conducted in 5 diverse districts, and methods included key informant interviews, focus groups, document review, health worker surveys, and analysis of service delivery data. Cycle 1 findings showed an uneven understanding of JKN regulations, unequal implementation readiness, and limited evidence of improved productivity. JKN capitation payments did not increase health worker satisfaction or motivate additional effort. Informed by these findings, regulations were rewritten and the capitation payment formula was redesigned to include payment conditional on performance. Cycle 2 found that health workers experienced increased workloads because of increased enrollment in JKN and the requirement that people access primary care before being referred to higher levels of care. In addition, health workers indicated they did not experience the payment system to be fair. Instead of payment being conditional on performance, they indicated it was primarily determined by education and tenure, with only some districts incorporating a small payment component based on behaviors, such as attendance, and performance. The health workers said they preferred to be paid based on achieving primary care targets. Conducting IR so that questions are relevant and the process of finding and sharing answers is timely and cost-effective requires high-level skills, but support to build IR capacity has potential to make a lasting impact.

## Abstract (precis)

Implementation research enabled stakeholders to formulate questions, assess implications of research results that informed changes in regulations and payment at the primary care level, and strengthen monitoring capacity. While the national health insurance system had some impact on performance of primary care facilities, individual providers remained unsatisfied because payment was largely based on factors outside of their control such as tenure and position, rather than their contributions to improved performance.

## BACKGROUND

Universal health coverage (UHC) reforms are complex and impact numerous processes, institutions, and individuals in health systems. To know whether reforms are being implemented as planned and working as envisioned, policy makers and managers need information and insights on bottlenecks. In Indonesia, the United States Agency for International Development (USAID) provided local capacity-building support to conduct implementation research (IR) to understand how health reforms were working for primary health care and to inform revisions. This article describes how IR was applied in Indonesia, shares lessons learned and trade-offs to consider when launching IR, and discusses why support for building IR capacity is a worthwhile investment.

IR in health can be defined as “a type of health policy and systems research concerned with the study of clinical and public health policies, programs, and practices, and aims to understand not only what is and is not working, but also how and why implementation is going right or wrong, and testing approaches to improve it.”^1^ IR focuses on practical and actionable issues and on complex and real-world settings. It involves implementers in shaping the research to meet their needs and relies on mixed methods to answer research questions. IR benefits both policy makers and implementers as a way to quickly identify and respond to implementation challenges by helping to answer questions such as:

- Is the initiative being implemented as planned?
- What factors are hampering implementation?
- Does the initiative translate into the expected changes in the system?
- Are there unintended consequences (either positive or negative)?
- What actions should be taken to improve implementation?

To our knowledge, IR has not been used in complex system-wide health reform efforts; however, we are aware of its application to scaling up health interventions^3^ and delivery strategies.^4^^,^^5^

In January 2014, Indonesia began implementing a national health insurance initiative aimed at covering the country's entire population by 2019. BPJS (Badan Penyelenggara Jaminan Sosial), Indonesia's social insurance administration organization, integrated existing insurance programs for the poor, civil servants, police, military, and formal sector workers into 1 single-payer national health insurance scheme, known as Jaminan Kesehatan Nasional (JKN). In addition, the vision was to integrate more than 300 district-level insurance schemes into JKN, and for all non-poor informal sector workers to enroll. If Indonesia attains these ambitious goals, it will have the largest single-payer insurance system in the world.

As with any ambitious health financing reform, implementation of JKN is challenging and not all effects can be anticipated. With its emphasis on actionable and prospective learning in real-world settings, IR can strengthen the chances of policy makers and implementers to successfully pursue universal health coverage. The purpose of the IR described in this article was to (1) strengthen local capacity in Indonesia to conduct IR and (2) provide crucial information for policy makers and other decision makers at the national and district levels about whether JKN was being implemented as intended and bringing about desired changes in primary care. Primary care was selected as the technical health area of focus because of its important to the entire health system. Policy and decision makers and managers can use the findings to monitor and strengthen operational processes, review policy decisions, and clarify roles and responsibilities to ensure progress toward reform and enable attainment of their goal to provide universal health coverage in Indonesia by 2019.

## METHODS

The research sites consisted of 5 districts in 4 provinces: East Jakarta (Jakarta Province), Jember (East Java Province), Tapanuli Selatan (North Sumatra Province), and Jayapura and Jayawijaya (Papua Province). These 5 districts exhibited strong local political commitment to JKN and were among USAID's priority districts for reproductive, maternal, and child health, tuberculosis, and HIV. They represented both urban and rural areas and were part of the Center for Health Policy and Management's (CHPM's) Health Policy Network, a network of universities across Indonesia. Before the research began, an assessment was conducted to review other studies on JKN and to identify gaps.

An important feature of the IR conducted in Indonesia was its participatory nature.^2^ CHPM and the Ministry of Health (MOH) Center for Health Finance and Insurance, supported by the Health Finance and Governance project team, facilitated an IR launch workshop with national- and district-level stakeholders in February 2016. The workshop used a root cause analysis method that unpacked the underlying processes and behavioral reactions that were expected to lead to anticipated benefits of JKN, which resulted in primary care as the priority focus of the IR.

Then, through a consultative process key stakeholders, including national and local policy makers and implementers, contributed to defining the IR questions, which broadly focused on the effects of JKN financing on primary care. Cycle 1 assessed how JKN regulations on capitation fund management at the primary health center level were being interpreted and implemented and implications for effectiveness of JKN. BPJS pays health centers a monthly per capita payment to deliver a package of services to JKN members. For public facilities, regulations mandate 60% of capitation funds for health staff supplemental payments and 40% for operational costs. Private primary care facilities have full discretion over how capitation funds are used. These capitation funds were hypothesized to motivate staff to provide improved primary care services, enhance productivity, and control costs by managing referrals. The operational costs component was hypothesized to improve availability of inputs and enhance outreach. Cycle 2 sought to investigate health worker satisfaction with capitation payments and identify opportunities to strengthen the links between capitation and behaviors that lead to improved service delivery.

Each cycle of research took approximately 1 year to complete and included engaging stakeholders, determining priority questions, launching field work, conducting analysis, and sharing results (Figure). Cycle 1 began in February 2015 and ended in December 2016, and Cycle 2 began during the Cycle 1 dissemination workshop in December 2016 and ended in December 2017. Specifically, to understand whether JKN capitation funds were being managed and used as intended, CHPM conducted focus groups and in-depth interviews with district health and government officials and health facility teams in the 5 target districts, complemented by document review, collection of administrative data on service delivery, and health worker satisfaction surveys (Table). Following the data collection and analysis phase in Cycle 1, stakeholders discussed the findings, identified whether and which corrective measures needed to be taken to improve implementation, and identified questions for Cycle 2.

**FIGURE.**
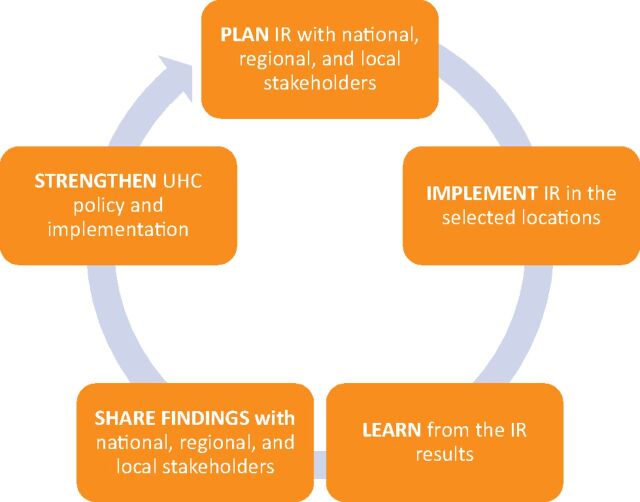
Implementation Research Cycle Abbreviations: UHC, universal health care; IR, implementation research.

**TABLE.** Number of Interviews and Focus Groups by Implementation Research Cycle and District

District	Cycle 1		Cycle 2	
	Interviews	Focus Groups (Participants)	Interviews	Focus Groups (Participants)
South Tapanuli	8	1 (15)	7	4 (29)
East Jakarta	26	1 (8)	15	4 (30)
Jember	24	1 (10)	18	4 (56)
Jayapura	15	1 (9)	14	6 (25)
Jayawijaya	13	1 (20)	9	3 (33)
Total	86	5 (62)	63	21 (173)

At the end of each of the 2 research cycles, the IR team at CHPM organized national- and district-level workshops to share findings and facilitate discussions with university partners and stakeholders. National-level decision makers valued learning about the challenges of implementing JKN from district leaders, and district leaders appreciated the opportunity to provide input to national decision makers. District leaders valued learning about how JKN was being operationalized in their communities and how other districts were interpreting and operationalizing JKN policies and regulations. Following national workshops, CHPM staff traveled to each district to meet with the local university partner and district stakeholders to discuss district specific findings.

### Partners and Stakeholders

The USAID-funded Health Finance and Governance Project used a competitive process to select the Center for Health Policy and Management (CHPM) at the University of Gadja Mada, to engage with stakeholders, carry out capacity building training, and conduct research. CHPM was selected because it had the following capabilities: (1) convening power and credibility to engage national- and district-level policy and decision makers to shape research and act on the findings, (2) capacity to learn from cycles of research that would strengthen JKN implementation at the primary care level, and (3) capacity to build local IR capabilities.

CHPM's government counterpart was the MOH Center for Health Finance and Insurance, which was chosen because they had a history of leading multi-entity working groups, had the mandate to focus on health financing and the impact of JKN on service delivery, had legal but not necessarily actual access to data from BPJS—the Indonesian entity that pays providers for services covered through the national insurance program, and they were receptive to engaging with CHPM to steer the consultation and dissemination process. Other stakeholders, selected because of national- and district-level leadership and policy and management roles, were the MOH Directorate of Primary Care, BPJS, Ministry of Home Affairs, Ministry of National Development Planning, and district health and political leadership.

### Strengthening Local Capacity in Implementation Research

CHPM conducted training sessions to build local capacity to conduct IR. CHPM trained 5 teams and 35 individuals from the staff and faculty of the Health Policy Network universities—University Sumatera Utera (North Sumatera Province), University Negeri Jember (East Java Province), and University Cendrawasih (Papua Province)—to conduct surveys, interviews, and focus groups; transcribe and analyze data; prepare briefs for district stakeholders; and present findings. Those trained both directly conducted the research and trained students and other faculty to conduct the implementation research. The capacity-building sessions enabled local individuals to conduct IR and communicate policy and programmatic findings across Indonesia.

In addition, CHPM conducted webinars and presentations on IR that reached 1,634 additional stakeholders comprised of district- and national-level decision makers, academics, and students.

## FINDINGS AND CORRECTIVE MEASURES

### Cycle 1

Findings from Cycle 1, assessing how capitation fund management regulations were being interpreted, revealed uneven understanding of national, provincial, and district regulations that impacted implementation of JKN at the primary care level. Another challenge identified was diverse readiness among districts and regions to manage JKN. For example, districts differed in their degree of use of capitation funds. Facilities in one district failed to use 36% of capitation funds because of imperfect understanding of procurement regulations, and in another district, facilities failed to use 17% of capitation funds because of challenges with procurement processes and under-spending for outreach.

Research findings revealed uneven understanding of JKN regulations, unequal implementation readiness, and limited evidence of improved productivity.

Staff surveys, interviews, and focus groups indicated that additional payments from capitation payments did not increase health worker satisfaction or motivate additional effort. Only 25% of doctors reported being satisfied with their income since introduction of JKN, whereas 43% reported being unsatisfied. One reason for doctor dissatisfaction was that additional payments received from capitation were not perceived to compensate for additional workloads, and another reason was that JKN forced them to work full shifts, which limited their ability to earn private-practice income.

The capitation payment system was found to have little impact on improving performance at either the facility level or the individual level. Findings from respondent interviews indicated that the additional income from capitation incentivized staff to show up for work, but there was limited evidence of improved performance. However, both quantitative and qualitative data indicated that preventive and promotive services increased in volume and quality.

In response to the finding of uneven understanding of regulations, the MOH held a meeting, together with the Ministry of Home Affairs, that was attended by 514 district health officers to understand the sources of misunderstanding, according to a conversation with Dr. Doni of the Health Finance and Insurance Unit of the Indonesia MOH (December 18, 2018). One outcome was that regulations were rewritten to be more user-friendly. Findings about weak incentive effect of capitation payments contributed to the redesign of the capitation payment to condition a portion on facility performance.

### Cycle 2

In 2017 the calculation for facility-level capitation payments was changed to a per capita basis as well as performance on 3 indicators: contact rate, referrals, and chronic disease management. Health promotion activities, including home visits, outreach services, exercise programs, and chronic disease care, increased due to the availability of funds to cover operational costs, including medicines and supplies, and potentially because of these new performance-based capitation payment rules. These changes contributed to improved ability to deliver priority services at the primary care level, but health workers remained unsatisfied. Our research sought to uncover the reasons why.

Findings from Cycle 2 showed that health workers did not find that additional income from capitation adequately compensated for the increased workload under JKN. The health workers perceived the allocation of payments to individuals to be unfair. Instead of health worker contribution to service delivery or population health, individual worker payments were primarily determined by immutable characteristics such as education attained, tenure, and position. Only some districts incorporated a small payment component that was determined by behaviors (e.g., attendance) and performance indicators (e.g., numbers of community visits and medical procedures).

The health workers indicated a preference for an incentive system based on service delivery accomplishments, such as measures of health promotion, preventive service delivery, and meeting quality standards. They also recommended adjusting payment for infectious disease risk and service area size. In addition to financial remuneration, health workers would like to be rewarded with opportunities for training and advancement. Finally, they recommended penalties for poor performance such as demotions, allowance reductions, and dismissal.

## LESSONS

It is not possible to comprehend how changes in systems, processes, payment rules, and roles and responsibilities are functioning without asking those who are affected. To answer many of those questions, a mixed-method approach is needed, which requires researchers to have both qualitative and quantitative skills. IR has the potential to be a powerful tool for understanding complex reform processes, but conducting IR so that the questions are relevant, the process of finding answers is cost-effective, and information is shared with policy makers so that timely action can be taken requires high-level skills.

External donors can support countries to build capacity to conduct IR and to use research findings to inform refinements. This support has potential to make a lasting impact that exceeds the tenure of any time-limited project. What follows are some lessons learned, challenges confronted, and trade-offs to consider.

### Communicating Controversial Findings

Because IR aims to understand what is and is not working smoothly, findings have the potential to be controversial. It was more effective to initially communicate IR findings through a series of one-on-one meetings than in group meetings with national and district stakeholders. Without advance preparation, some stakeholders were defensive in larger meetings. One-on-one meetings are time-consuming but we found them to be a necessary part of the process of ensuring that IR findings contribute to strengthening the health system.

Because implementation research findings can be controversial, one-on-one meetings were an effective first step to precede group meetings.

### Institutional Arrangements for Implementation Research

The institutional arrangements to conduct IR can impact its effectiveness. In Indonesia, an independent third-party university was selected to facilitate the IR process and to conduct the field research. The MOH was the key government stakeholder and convener of multi-stakeholder meetings on IR findings. However, both CHPM and the MOH reflected that it may be more effective for a government research unit to conduct IR directly or for the government to manage contracts with external entities to conduct this research, according to discussions with Dr. Laksono of CHPM (December 13, 2018) and with Dr. Doni of the Health Finance and Insurance Unit of the Indonesia MOH (December 18, 2018). One reason is that the MOH may be more receptive to findings that elements of the reforms are not working if the findings come from an internal unit. On the other hand, research conducted by a respected university may have more credibility than research conducted by a government team. The internal MOH research unit requested training on how to conduct IR from CHPM. In addition, a regulation was passed in 2018 that specifies that the internal MOH research group must be involved in all research in the health sector to ensure that research is policy relevant.

Deciding which key government stakeholders to include is not always obvious and selection may be influenced by external donor history. When support for IR began in Indonesia, USAID had a well-developed relationship with the MOH and a weaker relationship with BPJS. The MOH expressed interest in being a stakeholder and because they understood how to work with USAID external support, the MOH was selected as the key government partner. However, the role of BPJS as the strategic purchaser of health services solidified during the IR period, so there may have been a missed opportunity to focus the IR on whether key BPJS processes were functioning as intended.

### Capacity to Conduct Qualitative Research

Capacity to conduct qualitative research is critical for conducting IR that aims to understand how and why reforms may or may not be working at the service delivery level. Managing qualitative research in multiple districts with interviews and focus groups with multiple stakeholders generates volumes of transcripts that need to be coded and analyzed. Building capacity to conduct the qualitative research that is foundational to IR is a priority for low- and middle-income countries that aim to assess and refine the roll out of their UHC reforms.

Capacity to conduct qualitative research and manage complex field operations is critical to real-world monitoring through implementation research.

### Time to Institutionalize Implementation Research

Institutionalizing IR takes time. It takes time to identify the right stakeholders and to develop relationships with them and it takes time to prioritize questions and to implement fieldwork. This groundwork has begun in Indonesia and stakeholders are now discussing issues that were not the focus of conversations before the IR. A process of communication has been stimulated that cuts across national institutions and between national and district levels. The MOH has expressed interest in steering IR and has passed regulations requiring that research in the health sector must be policy relevant. While IR is in a nascent stage in Indonesia, policy makers, managers, and researchers recognize its value.

Implementation research is in a nascent stage in Indonesia, but policy makers, managers, and researchers recognize its value.

## CONCLUSION

Any country that introduces reforms to a complex health system needs to monitor whether processes are being understood and implemented as intended and whether the policies and systems are working as expected. In essence, this is sound management. It is “taking the temperature” of processes and systems so that there is regular information about aspects that need tweaking or changing. Leaders and managers operate with the hope that policies are working as intended, but IR supplies the information required to confirm that those hopes are realities.

## Notes

**Peer Reviewed**

**Funding:** USAID.

**First Published Online:** December 13, 2018

© Eichler et al. This is an open-access article distributed under the terms of the Creative Commons Attribution 4.0 International License (CC BY 4.0), which permits unrestricted use, distribution, and reproduction in any medium, provided the original author and source are properly cited. To view a copy of the license, visit http://creativecommons.org/licenses/by/4.0/. When linking to this article, please use the following permanent link: https://doi.org/10.9745/GHSP-D-18-00328
